# The determinants of individual health care expenditures in prison: evidence from Switzerland

**DOI:** 10.1186/s12913-018-2962-8

**Published:** 2018-03-07

**Authors:** Karine Moschetti, Véra Zabrodina, Tenzin Wangmo, Alberto Holly, Jean-Blaise Wasserfallen, Bernice S. Elger, Bruno Gravier

**Affiliations:** 1 Institute of Social and Preventive Medicine, University of Lausanne and University Hospital of Lausanne (CHUV), Route de la Corniche 10, 1010 Lausanne, Switzerland; 2 Technology Assessment Unit, University Hospital of Lausanne (CHUV), Lausanne, Switzerland; 3 Institute for Biomedical Ethics, University of Basel, Basel, Switzerland; 4 Institute of Health Economics and Management, HEC Lausanne, University of Lausanne, Lausanne, Switzerland; 5 University Centre of Legal Medicine, University of Geneva, Geneva, Switzerland; 6 Service of Correctional Medicine and Psychiatry, University Hospital of Lausanne (CHUV), Lausanne, Switzerland

**Keywords:** Prison, Health care expenditures, Outpatient care, Chronic diseases

## Abstract

**Background:**

Prison health systems are subject to increasing pressures given the specific health needs of a growing and aging prison population. Identifying the drivers of medical spending among incarcerated individuals is therefore key for health care governance in prisons. This study assesses the determinants of individual health care expenditures within the prisons of the canton of Vaud, a large region of Switzerland.

**Methods:**

We use a unique dataset linking demographic and prison stay characteristics as well as objective measures of morbidity to detailed medical invoice data. We adopt a multivariate regression approach to model total, somatic and psychiatric outpatient health care expenditures.

**Results:**

We find that chronic infectious, musculoskeletal and skin diseases are strong predictors of total and somatic costs. Schizophrenia, neurotic and personality disorders as well as the abuse of illicit drugs and pharmaceuticals drive total and psychiatric costs. Furthermore, cumulating psychiatric and somatic comorbidities has an incremental effect on costs.

**Conclusion:**

By identifying the characteristics associated with health care expenditures in prison, this study constitutes a key step towards a more efficient use of medical resources in prison.

## Background

Health care expenditures represent an increasing share of public spending and constitute a pressing concern in many developed countries. In correctional systems, these trends are likely to be exacerbated given the growth, aging, and specific health needs of the prison population [1, 2]. In Switzerland, the share of the population in prison has remained relatively stable at 85 individuals per 100,000 inhabitants in 2014, which is lower than the OECD average of about 150 [3]. However, the absolute number of incarcerated individuals increased by 25% between 2000 and 2014 [4]. As in most European countries, the principle of equivalence of care applies, meaning that all individuals in prison have the right to access the same standards of quality of health care as the general population [5–7]. Hence, prison systems are required to meet the health needs of individuals in prison with limited resources and while facing important organizational and ethical challenges.

The prison population has a high prevalence of chronic somatic and psychiatric conditions as well as substance abuse problems and infectious diseases [8–11]. Unmet needs prevail due to low socioeconomic status, precarious life experience, and limited access to health care prior to incarceration [12]. These factors contribute to explaining why health care utilization has been found to be greater in prison than the general population [13–19]. Furthermore, developed countries are witnessing an increase in the share of elderly prisoners [20, 21], which stems from harsher sentencing patterns with longer incarceration times [22, 23]. Older individuals in prison naturally have a higher prevalence of chronic health conditions, serious life-limiting illnesses and comorbidity rates [8, 11, 24]. Aging and chronic conditions are known to have major economic consequences on the health care system as a whole [25, 26]. In particular, non-communicable chronic diseases account for roughly 80% of total annual health care expenditures in Switzerland [27], with cardiovascular, musculoskeletal and psychiatric diseases being the largest burdens. Meanwhile, research lacks to understand the drivers of health care expenditures in prison. Empirical analyses have been hampered by the lack of individual-level data linking expenditures and clinical diagnoses. In light of the demographic trends and needs outlined above, understanding which factors are associated with individual health care expenditures represents a key step towards a more efficient use of resources and higher standards of care in prison.

This study investigates the determinants of outpatient health care expenditures in prisons. To this end, we estimate regression models for costs using a unique individual-level dataset from the canton of Vaud, a large region of Switzerland. We merge administrative data collected by prison medical staff with insurance invoice data for incarcerated individuals. The administrative data contain information on demographics and prison stay as well as a detailed profile of chronic diseases diagnosed by physicians. The invoice data capture all medical resources consumed by the individual within on-site prison outpatient clinics in 2011. This analysis complements previous studies that examine health care utilization in prisons [14, 17, 18, 28–32], none of which however consider costs. Our results show that costs are significantly associated with chronic infectious, musculoskeletal and skin diseases as well as schizophrenia, neurotic and personality disorders, and the abuse of illicit drugs and pharmaceuticals. Finally, we show that cumulating somatic and psychiatric comorbidities leads to a disproportional increase in costs.

## Methods

### Data description

This section presents the data, the cost outcomes of interest and the explanatory variables used to model them in the multivariate regression analyses. Methodological details on these analyses are provided in the next section.

#### Merged administrative and invoice data

Our analysis links two datasets. First, we use a cross-sectional dataset on 1664 adult individuals who were in a closed prison in the canton of Vaud at any point during the year 2011. The canton of Vaud has one of the largest prison systems in Switzerland with 641 spaces, that is about 9% of the total prison capacity in the country during that period [33]. The administrative data incorporates information on demographics, prison stay characteristics (e.g. mode of detention, length of prison stay), health care utilization, as well as a comprehensive set of objectively diagnosed chronic somatic and mental health conditions (see [11] for further details on this data). Each of the four studied prisons of the canton of Vaud has an on-site outpatient primary care clinic with nurses, generalist practitioners (GPs), psychiatrists and other visiting specialists, e.g. gynaecologists and physiotherapists. These clinics are the main points of provision of health care to individuals in prison and are operated by the Service of Correctional Medicine and Psychiatry (SMPP) of the University Hospital of Lausanne (CHUV). These data were systematically collected and updated by the medical staff of the SMPP. The Swiss legislation requires an examination by a GP within 3 weeks of being incarcerated to identify existing conditions and health needs. The data exclude individuals who did not have this routine medical examination upon incarceration and thus missing health data. They also exclude observations with missing values for any of the variables used in the empirical analysis.

Second, to obtain health care costs, invoice data were extracted from the accounting system of the CHUV, which centralizes all the bills sent to the health insurance companies for outpatient health care provided in prisons by the SMPP. In Switzerland, outpatient care is reimbursed through a fee-for-service system, so that invoices contain information on all health services provided and corresponding costs. The fees for outpatient services in prisons are the same as for the general population. The data also include expenses for all medications delivered on site.

These two datasets are matched to combine individual information with invoices. While non-matches are excluded, we retain 106 individuals who are reported not to have consumed health care in the administrative data and impute them as zero costs. These either had brief strays, or entered prison before the beginning of the year and then did not use health care. Concurrently, there are several explanations for some of the individuals who were reported to consume health care in the administrative data not matching with invoice data. The two datasets are collected independently, possibly leading to discrepancies and some bills not being issued. Sample selection issues related to the matching procedure are discussed below. The final analysis sample includes 1107 individuals.

#### Outcome variables: Total, somatic and psychiatric health care costs

As outcome variables, our regression analyses model three categories of outpatient health care costs obtained from invoice data. First, *total costs* include all expenditures from medical services used in the on-site outpatient prison clinics. Costs are cumulated when individuals have multiple prison stays in 2011. Second, we split total costs into somatic and psychiatric costs, corresponding to the two main types of health care provided in prison. This distinction allows to grasp the channel through which total costs are affected. The fee-for-service codes in invoice data are used to identify specific medical services and allocate them across somatic and psychiatric categories. *Somatic costs* comprise GP consultations, somatic care by nurses, somatic medication as well as physiotherapy, occupational therapy, and gynaecological consultations. Medical supplies and diagnostic tests are also assigned to somatic care. *Psychiatric* costs include consultations with psychiatrists, psychiatric care by nurses, ambulatory stays at the day psychiatric clinic and psychotropic drugs. We classify drugs as psychotropic if their Anatomical Therapeutic Chemical (ATC) classification code begins with N05 or N06, and as somatic otherwise [34].

#### Explanatory variables

The administrative data allow us to use a rich set of variables to model individual health care costs. Our regressions include binary indicators for chronic somatic diseases, mental health conditions and substance abuse problems (Table 2), categorized into groups according to the International Classification of Diseases, version 10 (ICD-10). This allows us to compare the magnitude and the significance of the influence of each disease group on costs. While some disease groups may drive up costs by requiring costly treatment with few contacts with physicians, others may remain relatively inexpensive despite requiring regular monitoring. Somatic diseases are diagnosed and reported by a GP, and include infectious diseases, skin problems, and diseases of the musculoskeletal, digestive, circulatory, endocrine, respiratory, and nervous systems. A psychiatrist reports psychiatric conditions and substance abuse problems. The former include schizophrenia, mood disorders, neurotic disorders, behavioural syndromes, personality disorders and mental retardation. The latter encompass illicit drug, pharmaceuticals and alcohol abuse.

Regression models also control for sex, age group, marital status and Swiss origin. A binary indicator for having health insurance acts as a proxy for socioeconomic status and prior access to health care. Basic health insurance is mandatory in Switzerland, so that all residents have to conclude contracts with private health insurance companies. These contracts are highly regulated and cover a wide range of medical services as determined by federal law. 1Contracts involve the payment of monthly premiums, and partial or full means-tested state subsidies exist to support low-income individuals. In particular, the insurance covers the costs of all health services provided in prison. For individuals with an existing health insurance contract, the premiums are financed using private funds whenever possible, or by state subsidies and contributions of the prison administration. However, more than half of the individuals in our sample are uninsured, mostly due to an illegal or highly vulnerable socioeconomic status (e.g. migrants, marginalized). This points to inequalities in access to medical care prior to incarceration for uninsured individuals, to whom prisons may offer an opportunity to access health care [35]. For these uninsured individuals, the prison administration directly bears the medical costs, or purchases basic health insurance on their behalf in case of longer sentences. Furthermore, in the canton of Vaud, the prison administration is completely legally and hierarchically separate from the prison health services, and does not intervene in medical decisions, with the exception of court-mandated psychiatric therapies. Prison health services do not pay medical costs but rather get reimbursed for all the care they provide, either by the prison administration or health insurance company. Hence, there should be no incentives for the prison medical staff to discriminate against individuals without health insurance, namely to make different treatment decisions based on insurance status. Moreover, healthcare service provision in Swiss prisons is governed by the medical and ethical guidelines of the Swiss Academy of Medical Sciences [36], under the principle of equivalence of care established by international norms [6, 37].

We capture prison stay characteristics by including indicators for the type of crime (sexual, drug-related or violent, with other as the baseline, e.g. traffic or fraud), number of stays in 2011 and an indicator for detention regime (preventive or convicted). We account for length of stay in 2011 to account for the fact that individuals with shorter stays have fewer opportunities to consume health care. Furthermore, total length of stay (including time spent in prison before 2011) may impact costs, since anxiety, isolation or withdrawal symptoms evolve over the incarceration time. Individuals with a shorter prison experience may consume health care more intensively than those who have stayed longer and had time to adapt. Concurrently, individuals with longer stays may need more care due to the psychological burden of long-term sentences, or conditions acquired in prison [18, 38]. We also include binary indicators for two specific types of sentences. First, individuals whose crime is related to severe mental disorders and high risks of recidivism are mandated psychiatric treatment under the Swiss Criminal Code (SCC, article 59) [39]. This measure can be extended for up to 5 years an unlimited number of times. Second, indefinite incarceration may also be ordered for serious offenders who are highly prone to recidivism, or are deemed untreatable (SCC, article 64).

Finally, prison facilities differ by types of detention regimes, capacity, social environment, and organization of medical care provision. To capture these differences, we include binary indicators equal to one if the individual stayed in a given prison and zero otherwise, considering that individuals may stay in more than one facility in a given year (e.g. transfers or multiple stays).

### Statistical analysis

#### Modelling health care costs

As outlined above, we use a multivariate regression approach to assess the determinants of the health care costs outcomes described in Section 2.2, using the explanatory variables presented in Section 2.3. Modelling health care costs poses several challenges due to the skewness, heavy-tails, and excess zeroes of their distribution. The performance of alternative regression methods has been widely tested in the econometric literature [40–46], but no single approach emerges as the optimal one [47, 48]. However, generalized linear models (GLMs) offer several advantages in this context. They avoid retransformation issues by estimating directly on the raw scale, thus accommodating zero costs and providing interpretable estimates^2^.

GLMs based on a linear exponential family density such as Poisson or Gamma can be estimated via pseudo-maximum likelihood, and provide consistent estimates as long as the link function (conditional mean) is correctly specified, even if the true density does not belong to the linear exponential family [49, 50]. Given this, the choice of the density only affects efficiency. However, as the link is likely to be misspecified to some extent, the fit will not be equally good over the whole range of predicted values [41]. GLMs may suffer from loss of precision with extremely heavy tails [40], as they impose restrictions on the whole distribution and do not allow to flexibly model higher order conditional moments [45, 51].

To select appropriate densities and link functions for our GLMs, we perform modified Hosmer-Lemeshow, Pregibon link, Pearson correlation, and modified Park tests [40, 47]. These tests work with raw-scale residuals and may be sensible to extreme values. We also conduct 50-fold cross-validation and compute mean prediction error (MPE), root mean square error (RMSE) and mean absolute prediction error, which measure the accuracy of individual predictions [44]. For conciseness, we only present results of selected tests at the bottom of Table 4. All models include the length of stay in 2011 as an exposure variable with a coefficient constrained to 1 to account for differences in opportunities to use care. Finally, robust sandwich standard errors are estimated to shield us against misspecifications of the variance [52, 53].

#### Average partial effects (APEs) of chronic health conditions and comorbidities

The next step is to determine the magnitude of the influence of specific chronic diseases and their combinations on costs in monetary terms. To this end, we use the GLM estimates to compute APEs using the method of recycled predictions. For each chronic disease *c* indicator, the APE is computed as

$$ {APE}_c=\frac{1}{N}{\sum}_i\left\{{\hat{\mu}}_i\left(\boldsymbol{x},1\right)-{\hat{\mu}}_i\left(\boldsymbol{x},0\right)\right\} $$

where ${\widehat{\mu}}_i\left(\boldsymbol{x},c\right)$is the predicted conditional mean of health care costs for individual *i* holding all other explanatory variables ***x*** constant. Specifically, we first predict costs for all individuals in the sample with the disease indicator switched off (equal to 0). Second, we predict costs for all individuals with the disease indicator switched on (equal to 1). Third, we average the differences in predictions across individuals. This approach provides an estimate of the average difference in costs from having the particular disease or not across all individuals and thus avoids covariate imbalance.

We also explore whether comorbidities have a mutually reinforcing influence on costs. In other words, we test whether the costs associated with having a given pair of conditions are greater than the sum of costs associated with having each disease separately. In particular, we focus on pairs of somatic and psychiatric diseases. Similarly to above, the additional cost of a comorbidity pair (*s*, *p*) is calculated as the average difference in APEs resulting from switching disease indicators on and off:

$$ {APE}_{sp}=\frac{1}{N}{\sum}_i\left\{{\hat{\mu}}_i\left(\boldsymbol{x},1,1\right)-{\hat{\mu}}_i\left(\boldsymbol{x},1,0\right)-\left[{\hat{\mu}}_i\left(\boldsymbol{x},0,1\right)-{\hat{\mu}}_i\left(\boldsymbol{x},0,0\right)\right]\right\} $$

where ${\widehat{\mu}}_i\left(\boldsymbol{x},s,p\right)$ is now estimated for combinations of the somatic condition indicator *s* and the psychiatric condition indicator *p*, holding other explanatory variables ***x*** constant.^3^

#### Sample selection

As outlined in Section 2.1, some individuals do not match across datasets. Hence, our estimates may be subject to sample selection bias if unobservable factors that influence the probability of matching are correlated with health care expenditures. We use the fully robust test proposed by JM Wooldridge [54] to investigate sample selection bias in regression models with log link. We refer the reader to the reference for further details on this test (p. 666).

## Results

### Sample and descriptive statistics

In the data merging procedure, out of the 1664 individuals present in the administrative data, 1107 (67%) individuals were matched to invoice data and enter our final sample, while 557 (33%) were not matched. However, the test for sample selection does not provide strong evidence for bias from dropping non-matched individuals (total costs *p*-value = 0.051; somatic costs *p*-value = 0.300; psychiatric costs *p*-value = 0.099).

Table 1 presents descriptive statistics of the explanatory variables for individuals in our final sample. The most prevalent chronic somatic conditions are infectious and musculoskeletal diseases. The prevalence of hepatitis B and C, HIV and tuberculosis is known to be high among incarcerated individuals [10, 11]. Musculoskeletal diseases (mostly back pain) may develop due to the uncomfortable conditions and lack of physical activity. Skin problems are also widespread in prison due to the confined environment [55]. Among psychiatric conditions, neurotic and personality disorders are most common. In terms of demographics, 47% of the sample consists of male individuals under 30 years old. Total incarceration length displays large variation and ranges from 1 day to 11 years in our sample.

**Table 1:** Descriptive statistics of chronic health conditions, demographics and prison stay characteristics

	Proportion in % (*N*)
Chronic somatic conditions	
Infectious diseases	9.2 (102)
Skin problems	6.4 (71)
Musculoskeletal system	10.7 (119)
Digestive system	7.3 (81)
Circulatory system	6.5 (72)
Endocrine system	3.4 (38)
Respiratory system	4.9 (54)
Nervous system	1.5 (17)
Psychiatric conditions	
Schizophrenia	2.4 (26)
Mood disorders	2.1 (23)
Neurotic disorders	16.0 (177)
Behavioural syndromes	1.7 (19)
Personality disorders	12.4 (137)
Mental retardation	2.4 (26)
Substance abuse	
Alcohol	9.8 (109)
Illicit drugs	17.5 (194)
Pharmaceuticals	5.7 (63)
Demographic characteristics	
Aged 18-29^a^	49.7 (550)
Aged 30–39	28.2 (312)
Aged 40–49	15.7 (174)
Aged 50 and older	6.4 (71)
Male^a^	92.3 (1022)
Female	7.7 (85)
Married	20.1 (223)
Swiss origin	17.5 (194)
Has health insurance	42.6 (472)
Prison stay characteristics	
Stayed in Bois-Mermet prison	37.6 (419)
Stayed in Plaine d’Orbe prison	14.4 (159)
Stayed in Croisée prison	50.5 (559)
Stayed in La Tuilière prison	11.6 (128)
Other type of crime^a^	69.3 (761)
Violent crime	2.8 (33)
Sexual crime	3.0 (31)
Drug-related crime	25.5 (282)
Convicted^a^	54.5 (603)
Preventive detention	45.5 (504)
Under SCC article 59	1.4 (16)
Under SCC article 64	0.3 (3)
Number of stays in 2011^b^	1.3 (0.6)
Total length of stay in 2011 in days^b^	97.5 (93.9)
Total incarceration time in days^b^	129.4 (239.3)

*N =* 1107. ^a^ Reference categories in regression models. ^b^ Mean (SD) for continuous variables. *SCC*: Swiss Criminal Code

Table 2 provides descriptive statistics of total health care expenditures across several subsamples. Somatic care represents roughly 30% of total costs, with almost 40% individuals having at least one chronic somatic condition. Psychiatric costs account for 70% of total costs and the proportion of individuals with mental health conditions is 40%. Individuals aged 50 and older are usually defined as elderly in prison, since they are in worse health than individuals of the same age in the general population [24]. Although these older individuals have higher somatic costs on average, they have lower total and psychiatric costs than younger individuals. Women cost more than men in all categories. Individuals with chronic somatic and psychiatric conditions have substantially higher expenditures in all categories. The average total cost for on-site outpatient care is of CHF 29 per individual per day of incarceration. These figures show the high degree of variation in individual costs. However, they are descriptive, and the observed relationships may be influenced by confounding factors, such as length of stay or health profile. The regression analysis in the next section provides evidence on the specific characteristics associated with costs.

**Table 2:** Descriptive statistics of health care costs by subsamples

	Cumulated costs (CHF)						Total cost per day of incarceration (CHF)		Length of stay in 2011 (days)	
	Total		Somatic		Psychiatric					
Sample (*N*)	Mean	(SD)	Mean	(SD)	Mean	(SD)	Mean	(SD)	Mean	(SD)
All (1107)	2217	(5416)	660	(2302)	1557	(4465)	28.73	(82.79)	97	(94)
Positive costs only^a^	2483	(5645)	749	(2439)	1734	(4679)	31.77	(86.51)	104	(93)
*Age group*										
Under 50 (1036)	2225	(5536)	645	(2351)	1580	(4580)	27.01	(67.48)	98	(94)
Aged 50 and older (71)	2105	(3225)	873	(1388)	1232	(2164)	53.80	(200.73)	95	(100)
Gender										
Male (1022)	2140	(5360)	598	(1891)	1542	(4589)	26.78	(77.91)	99	(95)
Female (85)	3142	(6008)	1402	(5069)	1740	(2542)	52.16	(125.91)	84	(75)
Chronic somatic conditions										
None (681)	1317	(3628)	307	(476)	1010	(3307)	21.06	(58.01)	83	(88)
One or two (345)	3496	(7393)	1083	(3091)	2413	(6160)	40.19	(114.26)	116	(97)
Three or more (81)	4339	(6342)	1823	(5199)	2516	(3579)	44.40	(93.22)	139	(105)
Psychiatric conditions or substance abuse										
None (672)	841	(2561)	492	(2325)	349	(724)	20.19	(91.28)	79	(87)
One or more (435)	4343	(7561)	919	(2243)	3425	(6650)	41.93	(65.54)	126	(98)
At least one somatic and one psychiatric condition or substance abuse (226)	5297	(8651)	1268	(2996)	4029	(7494)	43.13	(62.08)	139	(100)

^a^The means for total cost and length of stay only include the 1001 individuals with positive total costs. The means for somatic and psychiatric costs include 975 and 994 individuals with positive costs, respectively

### Results of cost regression models

Table 3 reports the exponentiated coefficients of Poisson and Gamma GLMs with log link for each cost outcome. These densities performed better overall in our selection tests, and are commonly used to model health care expenditures [42, 43]. The log link enables a direct interpretation of exponentiated coefficients as the multiplicative effect on the outcome of a unit change in the explanatory variable. Comparing these two models for the three cost categories allows us to test the sensitivity of results to the choice of the density. The modified Park test and the predictive accuracy measures favour the Poisson model for total and psychiatric costs, which also performs better than the Gamma GLMs in the other tests. The choice is less clear for somatic costs. The Hosmer-Lemeshow test has significant *p*-values in most cases, and the negative cross-sample MPE suggests that the models over-estimate expenditures on average, particularly in the upper end of the cost distribution^4^.

**Table 3:** Results of generalized linear models of total, somatic and psychiatric health care costs

	Total costs				Somatic costs				Psychiatric costs			
	Poisson-log		Gamma-log		Poisson-log		Gamma-log		Poisson-log		Gamma-log	
	Exp. coef.	(SE)	Exp. coef.	(SE)	Exp. coef.	(SE)	Exp. coef.	(SE)	Exp. coef.	(SE)	Exp. coef.	(SE)
Chronic somatic conditions												
Infectious diseases	2.749***	(0.411)	2.412***	(0.441)	6.323***	(1.405)	3.947***	(0.907)	1.848***	(0.268)	1.488**	(0.254)
Skin problems	1.493***	(0.231)	1.683**	(0.405)	1.979***	(0.430)	1.609***	(0.284)	1.221	(0.210)	1.604*	(0.450)
Musculoskeletal system	1.335***	(0.145)	1.758***	(0.289)	1.232*	(0.134)	1.680***	(0.223)	1.404**	(0.193)	2.020***	(0.407)
Digestive system	0.871	(0.107)	1.078	(0.131)	1.091	(0.149)	1.231*	(0.142)	0.755	(0.129)	1.025	(0.148)
Circulatory system	1.592	(0.531)	1.621*	(0.404)	2.546***	(0.904)	1.707***	(0.317)	1.047	(0.230)	1.447	(0.410)
Endocrine system	1.143	(0.286)	1.165	(0.258)	1.523	(0.414)	1.762***	(0.332)	1.072	(0.274)	0.973	(0.261)
Respiratory system	0.890	(0.142)	0.810	(0.109)	0.902	(0.170)	0.835	(0.102)	0.963	(0.171)	0.799	(0.122)
Nervous system	1.211	(0.279)	1.522*	(0.338)	1.184	(0.242)	1.155	(0.167)	1.139	(0.359)	1.737**	(0.482)
Psychiatric conditions												
Schizophrenia	2.549***	(0.468)	2.369***	(0.516)	0.967	(0.340)	0.748	(0.154)	3.090***	(0.648)	3.290***	(0.802)
Mood disorder	1.096	(0.197)	0.871	(0.156)	1.116	(0.309)	1.155	(0.246)	1.336*	(0.230)	0.854	(0.187)
Neurotic disorder	1.674***	(0.185)	1.622***	(0.173)	1.319*	(0.201)	1.466***	(0.166)	1.903***	(0.219)	1.776***	(0.201)
Behavioral syndrome	0.911	(0.170)	0.949	(0.172)	0.613**	(0.144)	0.587***	(0.103)	1.118	(0.232)	1.279	(0.277)
Personality disorder	1.455***	(0.190)	1.252*	(0.145)	1.304*	(0.177)	1.139	(0.136)	1.545***	(0.243)	1.326**	(0.170)
Mental retardation	1.261	(0.367)	1.189	(0.211)	1.355	(0.415)	0.810	(0.149)	1.197	(0.466)	1.460*	(0.308)
Substance abuse												
Alcohol	1.030	(0.119)	1.315***	(0.139)	0.871	(0.098)	0.952	(0.091)	1.054	(0.162)	1.573***	(0.191)
Illicit drugs	1.288**	(0.156)	1.878***	(0.269)	0.582***	(0.109)	1.060	(0.130)	1.710***	(0.239)	2.542***	(0.402)
Pharmaceuticals	1.914***	(0.277)	1.735***	(0.281)	1.559**	(0.288)	1.215	(0.185)	2.079***	(0.347)	2.219***	(0.479)
Demographic characteristics												
Aged 30–39	1.222*	(0.141)	1.372**	(0.173)	1.584***	(0.251)	1.153	(0.128)	1.056	(0.147)	1.508***	(0.213)
Aged 40–49	1.405***	(0.173)	1.397**	(0.184)	2.249***	(0.388)	1.521***	(0.206)	1.061	(0.146)	1.382**	(0.206)
Aged 50 and older	0.994	(0.287)	2.544***	(0.789)	1.377	(0.405)	1.811***	(0.405)	0.956	(0.275)	3.285***	(1.241)
Female	0.559**	(0.154)	0.809	(0.345)	1.207	(0.425)	1.157	(0.391)	0.374***	(0.105)	0.815	(0.403)
Married	1.144	(0.197)	1.146	(0.138)	1.152	(0.242)	1.011	(0.113)	1.085	(0.198)	1.133	(0.146)
Swiss origin	1.104	(0.134)	1.429**	(0.215)	0.817	(0.147)	1.166	(0.167)	1.240	(0.181)	1.602***	(0.265)
Health insurance	1.145	(0.123)	0.930	(0.097)	1.036	(0.185)	0.866	(0.085)	1.224	(0.162)	0.971	(0.115)
Prison stay characteristics												
Stayed in Bois-Mermet prison	0.558***	(0.090)	0.672**	(0.113)	0.798	(0.186)	0.766*	(0.121)	0.444***	(0.076)	0.620**	(0.126)
Stayed in Plaine d’Orbe prison	0.798	(0.136)	0.656**	(0.120)	0.989	(0.251)	0.785	(0.131)	0.685**	(0.130)	0.610**	(0.141)
Stayed in Croisée prison	0.840	(0.135)	0.783	(0.127)	1.086	(0.290)	0.910	(0.142)	0.687**	(0.112)	0.701*	(0.136)
Stayed in La Tuilière prison	1.621**	(0.347)	1.892***	(0.436)	1.619	(0.515)	1.428	(0.314)	1.344	(0.283)	1.764**	(0.455)
Violent crime	0.634*	(0.166)	0.919	(0.210)	0.743	(0.207)	0.869	(0.177)	0.713	(0.182)	1.035	(0.295)
Sexual crime	1.587**	(0.319)	1.411*	(0.279)	2.058**	(0.609)	1.449	(0.340)	1.478*	(0.306)	1.263	(0.291)
Drug-related crime	0.890	(0.106)	0.725***	(0.079)	0.973	(0.133)	0.824*	(0.083)	0.902	(0.109)	0.709***	(0.090)
Preventive detention	1.270**	(0.152)	1.204	(0.151)	1.366**	(0.203)	1.162	(0.131)	1.236	(0.166)	1.176	(0.161)
Under art. 59 SCC	3.009***	(0.755)	7.282***	(3.106)	1.372	(0.677)	2.580**	(1.152)	2.952***	(0.819)	9.796***	(4.406)
Under art. 64 SCC	1.340	(0.419)	4.216	(3.727)	0.714	(0.265)	0.960	(0.630)	1.620	(0.498)	6.678**	(6.303)
Number of stays in 2011	1.102	(0.150)	1.219*	(0.131)	1.100	(0.148)	1.056	(0.088)	1.138	(0.173)	1.305**	(0.168)
Total length of stay	0.999***	(0.000)	0.998***	(0.000)	0.998***	(0.000)	0.998***	(0.000)	0.999***	(0.000)	0.997***	(0.000)
Ln(Length of stay in 2011)	1 (exposure)		1 (exposure)		1 (exposure)		1 (exposure)		1 (exposure)		1 (exposure)	
Constant	11.427***	(1.883)	12.347***	(1.982)	2.522***	(0.658)	5.519***	(0.879)	9.225***	(1.636)	6.970***	(1.268)
Modified Park test^a^	1.032		1.459***		1.337***		1.724*		0.866		1.458***	
Hosmer-Lemeshow test (*p*-value)	0.068*		0.000***		0.000***		0.000***		0.091*		0.000***	
Cross-sample RMSE	4788.92		16,395.76		2305.08		2245.27		4474.69		24,926.08	
Cross-sample MPE	−82.91		− 1600.29		−16.97		−102.17		−116.94		− 2336.04	

*N =* 1107

Exponentiated coefficients with robust standard errors in parentheses. Aged 18–29, male, convicted and other type of offence are reference categories. Abbreviations: Swiss Criminal Code (SCC), root mean square error (RMSE) and mean prediction error (MPE)

* *p* < 0.10, ** *p* < 0.05, *** *p* < 0.01

^a^The table presents the estimate of the power of the conditional variance function, and significance levels indicate whether it is significantly different from the closest integer. For the Hosmer-Lemeshow test, *p*-values are presented

#### Chronic somatic conditions

Chronic infectious, skin and musculoskeletal diseases are associated with increased total costs in the Poisson model, and increased somatic costs in both GLMs. Having an infectious disease multiplies total costs by a factor of roughly 2.5, which is related to treatments being particularly expensive for these conditions. Circulatory diseases significantly increase somatic costs, while endocrine diseases are significant in the Gamma model only. Respiratory, digestive and nervous system diseases are not significant.

Interestingly, we also find a positive association between somatic diseases and psychiatric costs, which suggests that they partly capture the effect of psychiatric conditions or substance abuse problems. For example, individuals with psychiatric disorders and those who inject drugs may display more behavioural risk factors and comorbidity [35], making them prone to infectious diseases [56]. Out of the 102 individuals with infectious diseases in our sample, 59 have a substance addition, and 31 have a comorbid psychiatric disorder. These results point to an incremental cost of having both somatic and psychiatric diseases, which we explore further below.

#### Mental health conditions and substance abuse problems

Schizophrenia is a strong predictor of psychiatric and total expenditures, along with neurotic and personality disorders. Conversely, mood and behavioural disorders, which include diseases such as depression or anxiety, have coefficients below one or non significant for psychiatric and total costs. Behavioural syndromes are associated with significantly lower somatic costs. Psychiatric therapies are expensive, as they require face-to-face sessions with a specialist and costly psychotropic medication. The coefficients on illicit drugs and pharmaceuticals abuse are highly significantly positive for psychiatric and total costs. Substance addicts are often enrolled in methadone maintenance treatment programs managed by psychiatrists. Illicit drug abuse diminishes somatic costs in the Poisson model.

We also find positive associations between psychiatric disorders and somatic costs, which may have several explanations. Individuals with psychiatric disorders or substance abuse problems may be less autonomous in health self-management and particularly susceptible to violence and self-harm, thus increasing the need for consultations with GPs or nurses. Also, the use of psychotropic medication may induce metabolic side effects requiring somatic surveillance [57].

#### Demographic characteristics

Age is a strong predictor of costs in Gamma GLMs. Being aged 50 or older is associated with doubled total and somatic costs, and tripled psychiatric costs relative to the 18–29 year-old baseline. The pattern is less clear in Poisson models, which predict higher costs for individuals aged 30–39 and 40–49, but not for those aged 50 or older. Age is known to be positively associated with morbidity in both the general and the prison population [11]. Hence, the chronic disease indicators partially capture the influence of age on costs.

All else being equal, women have significantly lower total and psychiatric expenditures in Poisson models, despite their high prevalence of mental disorders and infectious diseases [11]. For somatic costs, the coefficients are above one but not significant. Previous studies find mixed evidence for women seeking more care relative to men in prison [14, 15, 28, 31]. However, they have underlined the specific needs of women in prison, which prison medical services are often not designed to satisfy. Marital status is not significant, while being of Swiss origin is associated with higher psychiatric and total costs in Gamma models. A possible explanation is that these individuals are more likely to have followed a therapy prior to incarceration, so that their conditions are more easily identified. Also, 44% of drug abusers and 63% of individuals with mandated psychiatric treatment are Swiss in our sample, so that these characteristics are correlated. These groups are prone to have a worse health status.

Insurance status is not a significant predictor of expenditures. This suggests that there is no discrimination in health care access across individuals in prison in terms of insurance status, and that having had easier access to health care prior to incarceration appears to have no direct impact on health care expenditures in prisons.

#### Prison stay characteristics

The total length of stay in prison is significantly negative, with an additional day in prison being associated with costs lower by 0.1–0.3%. This suggests that expenditures accumulate more slowly as the individual adapts to the prison environment. The number of stays does not affect costs. Having committed a violent crime displays no strong association, while crimes of a sexual nature are associated with larger total and psychiatric costs, as sexual offenders often follow a psychiatric therapy. Drug-related offences are negatively related to expenditures, but are significant in Gamma models only. Note that the characteristics of drug dealers may differ from those of consumers. Being mandated psychiatric treatment is associated with significantly higher total and psychiatric expenditures, while being incarcerated indefinitely displays no association. Individuals under preventive detention have higher costs, which may be explained by the shock of incarceration or the stress related to on-going judiciary proceedings. Their health status may also deteriorate due to them spending almost all day in their cell. Finally, estimations show that prison indicators are significant, which points to heterogeneity across facilities being correlated with health care costs.

### Average partial effects

Table 4 shows the APEs of chronic conditions on total costs expressed in Swiss francs (CHF), the scale of interest. Among the somatic disease groups, infectious diseases have the greatest APE and are associated with increases in total costs of about CHF 3200 to 4500. Schizophrenia is the costliest psychiatric disorder and induces expenditures between CHF 3100 and 4400. APEs vary across GLMs, with greater differences for diseases with large coefficients.

**Table 4:** Average partial effects of chronic health conditions on total costs (in CHF)

	Poisson-log GLM		Gamma-log GLM	
	APE	(SE)	APE	(SE)
Chronic somatic conditions				
Infectious diseases	3226***	(710)	4540***	(1448)
Skin problems	1054**	(479)	2390*	(1434)
Musculoskeletal system	711**	(292)	2496***	(966)
Digestive system	−291	(247)	280	(467)
Circulatory system	1272	(1125)	2089	(1364)
Endocrine system	315	(624)	582	(897)
Respiratory system	−245	(320)	− 695*	(419)
Nervous system	465	(609)	1865	(1217)
Psychiatric conditions				
Schizophrenia	3138***	(878)	4422***	(1548)
Mood disorder	210	(427)	− 469	(598)
Neurotic disorder	1308***	(318)	1920***	(501)
Behavioural syndrome	−197	(381)	− 183	(623)
Personality disorder	907***	(340)	836*	(440)
Mental retardation	569	(797)	670	(752.)
Substance abuse				
Alcohol	65	(260)	1076**	(480)
Illicit drugs	578**	(282)	2495***	(784)
Pharmaceuticals	1877***	(554)	2507**	(1009)

*N =* 1107. Standard errors calculated with the delta-method. Length of stay in 2011 (exposure) taken into account. * *p* < 0.10, ** *p* < 0.05, *** *p* < 0.01

Table 5 displays the additional expenditures generated by selected comorbidity pairs of chronic somatic and psychiatric diseases. Specifically, it presents the difference in the APE of each disease associated with also suffering from the other condition in the pair. The results suggest that it costs approximately CHF 4600 more to treat infectious diseases for individuals with schizophrenia compared to those without schizophrenia. Infectious diseases also have significant comorbidity costs in combination with the other psychiatric disorders. Neurotic disorders and pharmaceuticals abuse induce additional costs when cumulated with skin or musculoskeletal diseases. Circulatory diseases do not generate comorbidity costs with psychiatric conditions. These results are in line with previous evidence for the general population showing that the incremental cost of an additional chronic disease increases with the number of existing conditions [58].

**Table 5:** Average partial effects of selected comorbidities on total costs (in CHF)

	Infectious diseases		Skin problems		Musculoskeletal diseases		Circulatory diseases	
	APE	(SE)	APE	(SE)	APE	(SE)	APE	(SE)
Psychiatric conditions								
Schizophrenia	4576***	(1681)	1487*	(883)	1005*	(536)	1795	(1452)
Mood disorder	306	(613)	100	(211)	67	(136)	121	(236)
Neurotic disorder	1893***	(607)	621**	(316)	419**	(210)	753	(623)
Behavioral syndrome	−287	(552)	−94	(180)	−63	(126)	−113	(247)
Personality disorder	1318**	(586)	431	(286)	291*	(164)	521	(449)
Mental retardation	827	(1166)	271	(383)	183	(254)	327	(541)
Substance abuse								
Alcohol	95	(379)	31	(126)	21	(83)	37	(149)
Illicit drugs	853**	(372)	275	(180)	185*	(109)	331	(303)
Pharmaceuticals	2750**	(1017)	894**	(427)	602**	(305)	1075	(1058)

*N =* 1107. Standard errors calculated with the delta-method. Length of stay in 2011 (exposure) taken into account

* *p* < 0.10, ** *p* < 0.05, *** *p* < 0.01

## Discussion

This study explores the drivers of individual health care expenditures for outpatient care within the prisons of the canton of Vaud, Switzerland. We construct a unique individual-level dataset by combining detailed invoice data with a wide set of objective measures of morbidity as well as demographic and prison stay information. We run regression models to assess the magnitude and significance of the associations between these characteristics and total, somatic and psychiatric health care expenditures. Furthermore, calculating the APEs allows the estimation of the economic impact of chronic conditions as well as the additional costs of cumulating psychiatric and somatic comorbidities.

This paper adds to the scarce literature on the health profile and health care utilization of incarcerated individuals.

The results provide key insights into the costs of medical resources provided in prison, and inform the allocation of scarce financial resources in prisons by identifying individual characteristics and health conditions that predict higher expenditures. A more efficient identification of individuals who present these characteristics may significantly improve their management and outcomes, and subsequently lead to lower expenditures. In our sample, less than 100 individuals induce more than half of the total on-site outpatient costs. From the perspective of the prison administration, the results provide relevant information to identify those individuals, for whom health insurance should be purchased rapidly.

Among somatic diseases, we identify chronic infectious diseases, musculoskeletal and skin problems as important predictors of health care expenditures within prisons. Infectious diseases in particular remain a serious issue in correctional facilities and an important target for prevention. Schizophrenia, neurotic and personality disorders as well as illicit drugs and pharmaceuticals abuse are significantly associated with increased total and psychiatric costs. Furthermore, individuals cumulating somatic and psychiatric conditions induce additional costs directly related to the comorbidity itself. Hence, the high predisposition of individuals with psychiatric and substance abuse disorders towards risky health behaviours make them a particularly pertinent target group. Psychotropic treatments may also cause somatic side effects that are difficult to manage in prisons. These considerations underscore the importance of coordination between psychiatric and somatic health care professionals. More generally, the incremental costs of comorbidities are an increasingly relevant issue both in prisons and in the general population.

Prison infrastructures and health systems are often not designed to manage somatic chronic conditions efficiently, which may exacerbate their impact [59]. Regular follow up consultations with nurses and GPs are complicated by security and logistic concerns, adding indirect costs to those of medical care. The need for consultations is increased in prison since, unlike the general population, incarcerated individuals cannot benefit from informal health care contacts, e.g. with relatives or pharmacists [15, 16]. In this context, prevention can contribute to avoid developing or worsening of chronic illnesses – along with costly complications and comorbidities – and to contain costs. In particular, fostering health literacy and informing individuals in prison on how to manage their chronic somatic conditions may increase treatment adherence and outcomes, while reducing the need for medical contacts. Additionally, ensuring continuity of treatment both outside of the prison and throughout the incarceration period may favour reintegration and socioeconomic stability upon release, as well as decrease the probability of costly acute complications.

An interesting feature of the environment under study is the absence of inequalities in access to health care. Indeed, the medical services offered in prison are relatively standardized, and the institutional setting eliminates the direct role of financial enabling factors, since incarcerated individuals incur no out-of-pocket costs. This differs from the general population, which typically faces inequalities in access to and quality of care related to socioeconomic status. However, individuals in prison have specific needs and therefore are not a representative subgroup of the general population. For example, individuals in prison may consult medical staff because of difficulties to adjust to the correctional environment, a wish to relieve boredom, or the hope to obtain psychotropic drugs, which have resale value within prisons. Organizational constraints also exist that may create a wedge between the desirable and actual levels of health care utilization. Finally, the absence of provider choice in prison and competition mechanisms could limit incentives to improve quality of care and contain costs.

### Limitations and strengths

This analysis has several limitations. First, with regards to captured costs, our data do not include costs generated by off-site health care services, namely specialized outpatient care unavailable on-site, emergency admissions or inpatient care. Complete cost data were not available for these services since providers other than the CHUV may supply them. Off-site care is typically costly, primarily because concerned individuals require emergency admissions or hospital stays and are usually more severe. Off-site transfers also involve complex security measures and generate further non-medical expenses. However, off-site care complements rather than substitutes regular outpatient care in prison clinics, since prison medical staff act as gatekeepers for off-site care and are responsible for subsequent follow ups. Not accounting for these off-site is thus unlikely to deflate (or shift) the on-site costs. Furthermore, on-site outpatient care represents more than 95% of the total number of outpatient consultations, making this study highly relevant for prison health care governance. Further research aiming at evaluating individual risk factors for off-site health care utilization would be pertinent.

As for the on-site outpatient care under study, it is possible that some services were not entered into the accounting system, so that costs may be underestimated. In particular, short visits to nurses for routine blood pressure level checks or medication administration may have been overlooked. These are likely to represent low costs, and there is no reason to believe that billing depends on any individual characteristics so as to bias our estimates. Another limitation of our data and hence our models is that we do not include acute episodes such as influenza or hunger strikes. However, chronic conditions generate a substantial part of these acute complications (e.g. blood sugar drops for individuals with diabetes, or self-harm for individuals suffering from depression). Finally, data are available only for one region of Switzerland, which limits external validity.

## Conclusion

Prisons play a crucial role in addressing the medical needs of incarcerated individuals, whose access to health care prior to detention is often limited. Most of them are eventually released, so that poorly managed health problems may additionally burden the health care system outside the prison [12]. In light of the high disease prevalence even in young prison populations, the pressures on prison health systems are bound to increase as the population grows and ages. While this study provides insights into the patterns of individual health care expenditures in prison, further evidence on the cost-effectiveness and organization of prison health care provision is key to ensure adequate quality of care for incarcerated individuals as well as working conditions for prison staff. Data availability in correctional settings remains a challenge to progress in this area.

## Abbreviations

APE: Average partial effect
ATC: Anatomical Therapeutic Chemical Classification
CHUV: University Hospital of Lausanne (Centre Hospitalier Universitaire Vaudois)
GLM: Generalized linear model
GP: General practitioner
ICD: International Classification of Diseases
MPE: Mean prediction error
RMSE: Root mean squared error
SCC: Swiss Criminal Code
SMPP: Service of Psychiatry and Correctional Medicine (Service de Psychiatrie et Médecine Pénitentiaire)

## Footnotes

1 For further details on the Swiss health and insurance systems, see e.g., the review published by the OECD and the WHO [60].

2 Ordinary least squares of log-transformed expenditures are commonly used and may be efficient with heavy-tailed data [40]. However, this method poses the retransformation problem to obtain interpretable results, especially in the presence of heteroscedasticity, and is inappropriate for zero data. Breusch-Pagan and White tests detect complex heteroscedasticity of log-scale residuals, and the Shapiro-Wilk test rejects normality (all *p*-values < 0.000).

3 We experimented with models including interaction terms between disease indicators, but these led to severe collinearity issues.

4 Plotting the mean residuals by deciles of costs indicates that the models consistently over-predict in the upper decile. The Gamma model in particular downweighs the errors at the high end of costs compared to the Poisson model, which gives equal weight to errors across the whole range. Therefore, the Gamma fits high-cost observations worse [41].
